# Characterization of chemical reactions of silver diammine fluoride and hydroxyapatite under remineralization conditions

**DOI:** 10.3389/froh.2024.1332298

**Published:** 2024-03-01

**Authors:** M. Kaur, S. Shahid, N. Karpukhina, P. Anderson, F. S. L. Wong

**Affiliations:** Dental Physical Sciences Unit, Centre for Oral Bioengineering, Institute of Dentistry, Faculty of Medicine and Dentistry, Queen Mary University of London, London, United Kingdom

**Keywords:** remineralization, MAS-NMR, cariostatic, SDF, silver chloride, fluoride-substituted hydroxyapatite

## Abstract

**Introduction:**

Silver Diammine Fluoride (SDF) is a clinically used topical agent to arrest dental caries. However, the kinetics of its chemical interactions with hydroxyapatite (HA), the principal inorganic component of dental enamel, are not known. The aim was to characterize the step-wise chemical interactions between SDF and HA powder during the clinically important process of remineralization.

**Methods:**

Two grams of HA powder were immersed in 10 ml acetic acid pH = 4.0 for 2 h to mimic carious demineralization. The powder was then washed and dried for 24 h and mixed with 1.5 ml SDF (Riva Star) for 1 min. The treated powder was then air-dried for 3 min, and 0.2 g was removed and stored in individual tubes each containing 10 ml remineralizing solution. Powder was taken from each tube at various times of exposure to remineralization solution (0 min, 10 min, 2 h, 4 h, 8 h, 24 h, and 10 days), and characterized using Magic Angle Spinning-Nuclear Magnetic Resonance (MAS-NMR) spectroscopy.

**Results and discussion:**

^19^F MAS-NMR spectra showed that calcium fluoride (CaF_2_) started to form almost immediately after HA was in contact with SDF. After 24 h, the peak shifted to −104.5 ppm suggesting that fluoride substituted hydroxyapatite (FSHA) was formed with time at the expense of CaF_2_. The ^31^P MAS-NMR spectra showed a single peak at 2.7 ppm at all time points showing that the only phosphate species present was crystalline apatite. The ^35^Cl MAS-NMR spectra showed formation of silver chloride (AgCl) at 24 h. It was observed that after the scan, the whitish HA powder changed to black color. In conclusion, this time sequence study showed that under remineralization conditions, SDF initially reacted with HA to form CaF_2_ which is then transformed to FSHA over time. In the presence of chloride, AgCl is formed which is subsequently photo-reduced to black metallic silver.

## Introduction

Dental caries is a multifactorial process leading to a net mineral loss of dental hard tissues. It is a dynamic process which depends on the interaction of protective and pathologic factors in saliva and plaque biofilm (1). As reported in the global survey (2), 2.3 billion people suffer from dental caries of permanent teeth and more than 530 million children suffer from caries of primary teeth. In the UK, dental caries is the most common preventable disease and despite the prevention procedures provided by dentists in UK, the prevalence of experience of dental decay in 5-year-old children in England (d3mft) was 23.4%.

In the oral environment, the caries process is an alternating cycle of demineralization, the loss of tooth mineral tissue (principally calcium hydroxyapatite) via reactions with organic acids at lower pHs, and remineralization the redeposition of mineral from local calcium and phosphate ions at higher pHs (3) leading to net loss of mineral from the tooth, resulting in cavitation (1). If demineralization exceeds remineralization, then tissue loss occurs, whereas, if remineralization exceeds demineralization, then tissue replacement occurs, which is the aim of non-surgical clinical intervention such as SDF. Saliva is a unique biologic fluid with a complex composition. Saliva acts as a buffering agent, and plays an important role in the demineralization and remineralization in the oral cavity. Salivary calcium, phosphorous and hydroxyl ions are at a dynamic equilibrium with apatite mineral in enamel (4). During remineralization, the calcium and phosphate ions combine with the fluoride ions to rebuild a new surface layer on the subsurface demineralized lesion (1).

Non-restorative caries control (NRCC) treatment with silver diammine fluoride (SDF) is becoming a popular management strategy (5, 6). In the UK (for example), during the COVID pandemic, SDF was used as an intervention to arrest/remineralize cavitated carious lesions in primary teeth for pre-cooperative children due to the long general anesthetic waiting list (7). Clinical trials showed that SDF is an effective cariostatic agent, and safe to be used in children (6–14). However, SDF has the disadvantages of staining teeth black, unpleasant taste, gingival burn, and tattooing, which deters dentists to use it routinely due to low parental acceptance (15).

Solid state Nuclear Magnetic Resonance (NMR) spectroscopy is used to characterize compounds formed in chemical interactions and has been used in inorganic mineralized tissue dental research to identify various components (16, 17). For example, ^19^F Magic angle spinning (MAS)-NMR can identify all existing fluorine compounds in crystalline, amorphous, or adsorbed forms, within enamel mineral (18–20).

It is known that fluoride (F) interacts with the hydroxyapatite (HA) in enamel or dentine to form fluorapatite (FA) which provides cariostatic protection (e.g., 19, 21–23). Further, other studies have investigated the compounds formed when high concentration F products such as SDF (44,800 ppm F) interact with dental hard tissues (19, 24, 25). However, these previous studies did not investigate the intermediate phases in a time sequential manner, or used the detailed capability and sensitivity of ^19^F MAS-NMR. Hence, the aim of this current study was to investigate the chemical interactions between SDF and HA powder, and characterize the products, under standard *in vitro* remineralizing conditions at a sequence of time points within 24 h (known to be the time period over which the calcium is used up) and finally at 10 days using ^19^F, ^31^P and ^35^Cl MAS-NMR spectroscopies in order to understand the complex chemistry during the remineralization processes.

## Materials and methods

To mimic exposure of dental hard tissue mineral to cariogenic acidic conditions, 2 g of HA powder (4.14 µm particle size, P3R SD, Captal HA, Plasma Biotal, UK) were immersed in 10 ml of demineralizing solution (0.1 mol/L acetic acid buffered to pH = 4.0 using potassium hydroxide) in a centrifuge tube and placed in a shaking-incubator at 37˚C for 2 h (26, 27). After centrifugation for 3 min, the powder was collected, washed, and dried on filter paper for 24 h in an incubator at 37°C. The demineralized HA powder was then mixed with 1.5 ml of 38% SDF (Riva Star, SDI, Australia, LOT 1213678) solution for 1 min, using cement spatula and made into a paste and then air-dried for 3 min, following the British Society of Paediatric Dentists (BSPD) clinical protocol for SDF application. The SDF treated demineralized HA powder was divided equally into 10 samples of 0.2 g each and stored in darkened centrifuge tubes to prevent light interaction with SDF. In 7 of the tubes, 10 ml of remineralization solution [2.0 mmol/L CaCl_2,_1.2 mmol/L KH_2_PO_4_, 150 mmol/L NaCl and buffered to pH = 7.0 using potassium hydroxide; as described by Siddiqui et al. (28)] were added. These tubes were placed in a shaking incubator at 37°C for different time intervals (*t* = 0 min, 10 min, 2 h, 4 h, 8 h, 24 h, and 10 days). At the end of each time point, the powder was collected from one of the tubes, washed, dried and analyzed using MAS-NMR spectroscopies.

### MAS-NMR spectroscopy

^19^F, ^31^P and ^35^Cl MAS-NMR spectra were collected using a 600 MHz, 14.1 T, Avance NEO spectrometer (Bruker, Germany) using the parameters listed in Table 1. The ^35^Cl MAS-NMR spectra were referenced to 0 ppm of the signal in solid NaCl purchased commercially (29, 30). The spectra were processed and analyzed using the TopSpin software package (Bruker, version 4.0.8).

**Table 1:** Parameters used for MAS-NMR.

Parameters for MAS-NMR	^19^F	^31^P	^35^Cl
Resonance frequency (MHz)	564.8	242.9	58.8
Spinning frequency (KHz)	22	12	12
Signal of reference adjusted chemical shift/ppm	−120	0	0
Number of scans	128	32	512
Size of rotor	2.5mm	4mm	4mm
Reference material	1 mol/l aq NaF	85% aq H_3_PO	Solid NaCl

## Results

**Figure 1:**
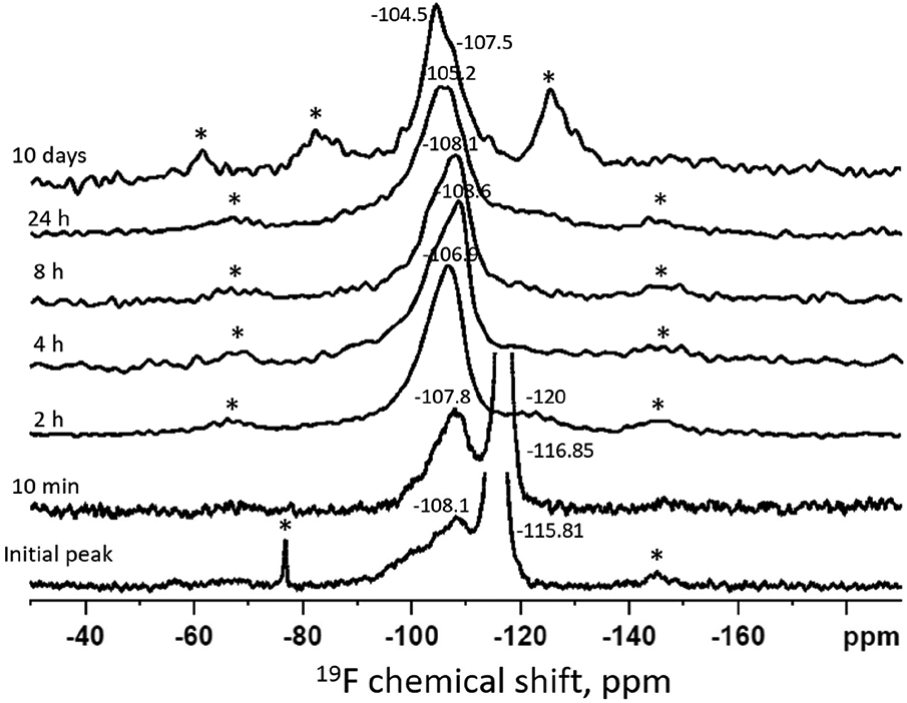
Time series of ^19^F MAS-NMR spectra of HA powder treated with SDF and immersed in remineralization solution. The duration of immersion is indicated next to each spectrum. The asterisks show the spinning side bands. The initial spectrum shows a peak position at −116 ppm indicating presence of free fluoride and the broad peak at −108 ppm suggesting the presence of a mixture of CaF_2_ and FSHA. With passage of time, the peak shifted from −108 ppm to −104.5 ppm indicating that more FSHA were formed at the expense of CaF_2__._

Figure 1 shows the time series of ^19^F MAS-NMR spectra of HA powder treated with SDF and immersed in remineralization solution. The initial (*t* = 0 min) spectrum shows a dominant sharp peak at −115.8 ppm which is demonstrative of loosely bound fluoride adsorbed on the surface (31). This sharp signal was also present in the *t* = 10 min sample, though with the center shifted to −116.9 ppm. Also, the broad minor peak centered at −108.1 ppm shows instantaneous reactionary products. Similarly, at *t* = 10 min, a very small and broad peak was visible at −107.8 ppm indicating the formation of calcium fluoride (CaF_2_) (18). At *t* = 2 h, the sharp peak around −116 ppm was replaced by a broad peak at −107 ppm confirming formation of CaF_2_ (18). At *t* = 4 h, CaF_2_ formation continued as indicated by the broader peak at −108 ppm. At *t* = 24 h this peak position shifted to −105.2 ppm, indicating the formation of fluoride substituted hydroxyapatite (FSHA) (18), which is a mineral in which some (but not all) of the hydroxyl (-OH) groups in HA are substituted by F. At *t* = 10 days, the broad peak remained but shifted to −104.4 ppm, confirming the formation of FSHA. In addition to this signal, the spectra at *t* = 24 h and *t* = 10 day also showed a peak at −108 ppm.

**Figure 2:**
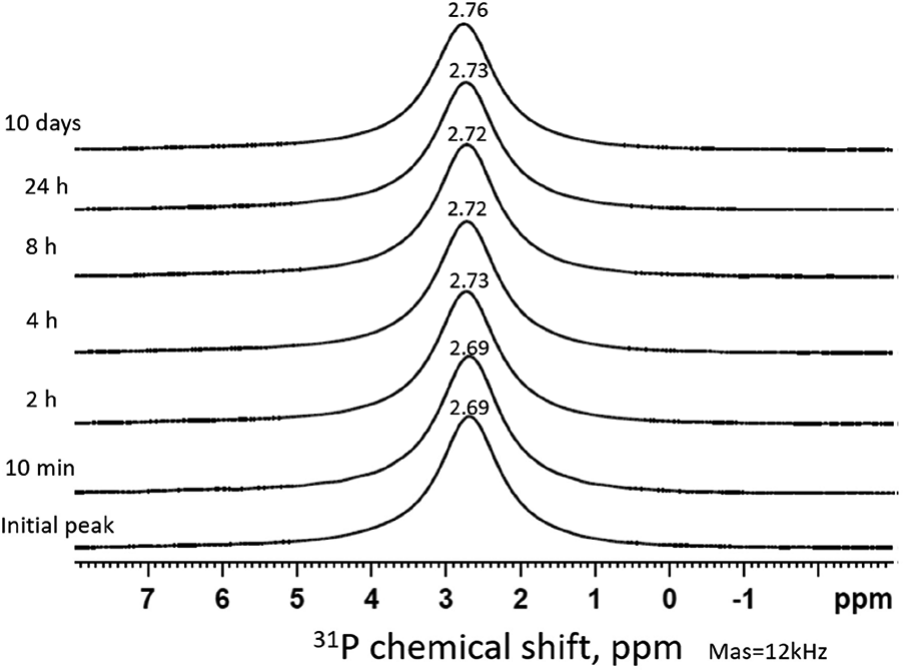
Time series of ^31^P MAS-NMR spectra of HA powder treated with SDF and immersed in remineralization solution. The duration of immersion is indicated next to each spectrum. The peak position (2.7 ppm) represents the HA pattern which remains the same throughout the time sequence.

Figure 2 shows the time series of ^31^P MAS-NMR spectra of the demineralized HA powder treated with SDF, collected after immersion in remineralization solution. From *t* = 0 to t = 24 h, there was only one single sharp peak around 2.7 ppm, suggesting the crystalline structure of the HA did not change during their exposure to remineralization solution. No other phosphate phases were detected.

**Figure 3:**
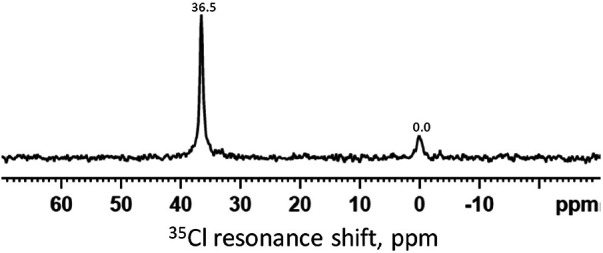
^35^Cl MAS-NMR spectrum of the demineralized HA powder treated with SDF and immersed in the remineralization solution for 24 h. The peak at 0 ppm is the reference peak for NaCl. The peak at 36.5 ppm indicates the presence of AgCl.

Figure 3 shows a ^35^Cl MAS-NMR spectrum of the demineralized HA powder treated with SDF after immersion in remineralization solution for 24 h. The spectrum showed the reference peak at 0 ppm for NaCl. The sharp peak at 36.5 ppm shows presence of silver chloride (AgCl).

**Figure 4:**
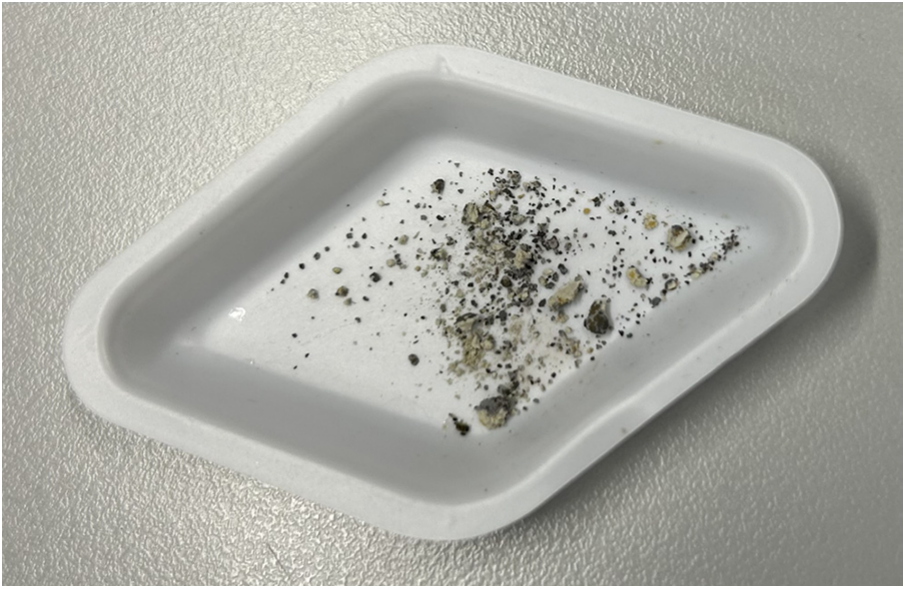
Powder retrieved after NMR scan and exposed to light. The white powder turned to black indicating metallic silver was formed.

After the NMR scan, when the powder was retrieved, the whitish color changed to black as shown in Figure 4.

## Discussion

The ^19^F MAS-NMR results (Figure 1) shows CaF_2_ was dominantly formed at an early stage (*t* < 2 h). This could be due to the very high F concentration (44,800 ppm) in SDF. When SDF dissolved in the remineralizing solution, the free F^−^ ions could react rapidly with the Ca^2+^ ions in the solution to form insoluble CaF_2_ (20, 32, 33). However, as the signal at −108 ppm was broad and covered a wide range of values with down to −100 ppm, contemporaneous formation (albeit a small amount) of FSHA (between −102 and −107 ppm) at this initial state cannot be excluded. Furthermore, the asymmetrical peaks at −106 to −104 ppm at later time points indicated the overlap of FSHA and CaF_2._ However, no fully fluoride substituted fluorapatite (FA) peak was observed. Investigating the chemical shift of the current spectra, the maximum substitution was up to 20% (18). From the trend of the chemical shift, FSHA was formed over time at the expense of CaF_2._ In the oral environment, the SDF may interact saliva with high calcium rapidly to form insoluble CaF_2_, which acts as a reservoir for FSHA formation, providing protection against acidic attack, though not as effective as fully substituted FA (34–36).

The ^31^P spectra (Figure 2) show the presence of HA in all time points, mainly from the HA powder. It is surprising that no other phosphate products such as silver phosphate (Ag_3_PO_4_) was detected, as reported in previous literature (26, 37). This is due to the presence of NaCl in the remineralizing solution, causing the formation of AgCl instead (Figure 3). As the content of Ag^+^ was small compared to the NaCl concentration, all the Ag ions were used up before they could combine with the phosphate ions. In previous studies, the demineralizing solutions did not contain NaCl, hence, Ag_3_PO_4_ was formed (26).

In the present experiment, the powders removed from the tubes were white as they were kept away from light. The black color (Figure 4) after NMR scan was likely due to the photo-reduction of AgCl to metallic silver. Clinically, SDF is topically applied using an applicator brush onto carious tooth surfaces which turn black in minutes, mainly on dentine and less so on enamel. As oral environment saliva contains chloride ions, it is likely AgCl particles are formed, which is a whitish insoluble powder. If the AgCl particles are deposited on the smooth enamel surface, they will be washed away. However, if they are deposited and accumulate in rough exposed dentinal tubules, they cannot be washed away quickly. The AgCl is then photo-reduced to black metallic silver which causes the discoloration in dentine. As these insoluble Ag particles block the dentine tubules, they may act as pulpal barrier, thus reducing dental pain and have anti-bacterial effect to reduce caries progression (38–40).

## Conclusions

This study characterized the products formed as a reaction between HA and SDF under remineralizing conditions. It was found that initially CaF_2_ was formed, which subsequently changed to FSHA over a 24 h period. AgCl was formed rapidly which could be photo-reduced to metallic silver.

## Data Availability

The raw data supporting the conclusions of this article will be made available by the authors, without undue reservation.
